# Patients with depressive disorder, their co-morbidity, visiting rate and disability in relation to self-evaluation of physical and mental health: a cross-sectional study in family practice

**DOI:** 10.1186/1471-2296-10-38

**Published:** 2009-06-01

**Authors:** Kadri Suija, Ruth Kalda, Heidi-Ingrid Maaroos

**Affiliations:** 1 Department of Polyclinic and Family Medicine, University of Tartu, Puusepa 1a 50406, Tartu, Estonia

## Abstract

**Background:**

High prevalence of depression among primary care patients has increased the need for more research in this field. The objectives of our study were to analyse how depressed patients evaluate their health; which co-morbid diseases are associated with depression; how depression influences the patients' consultation rate in family practice (FP); how disability is associated with depression; and how depression influences the patients' working ability.

**Methods:**

A cross-sectional study, part of the PREDICT study. The study group was formed of 1094 consecutive patients from 23 FPs across Estonia, aged 18–75 years, attending a FP to consult the family doctor (FD). Occurrence of major depression during six months was estimated using the Depression Section of the Composite International Diagnostic Interview. The medical records of all patients were analysed concerning co-morbid diseases, number of visits to the FD, and disability. Every patient filled in questionnaires to assess health-related risk factors for depression, and the SF-12 Health Survey to assess functioning and the perception of health.

**Results:**

Depression was found in 230 (21%) of the patients. Depressed patients reported less accomplishment owing to emotional problems (OR 1.80; 95% CI 1.18–2.72), being less careful as usual (OR 1.81; 95% CI 1.26–2.60), and having pain that extremely interfered with their normal work (OR 2.50; 95% CI 1.33–4.70) in comparison with non-depressed patients. Also depressed patients were more days on sick-leave (OR 1.00; 95% CI 1.00–1.01) than non-depressed patients. However, analysis of the medical records did not indicate that depressed patients consulted the FD more or had more co-morbid diagnoses than the non-depressed patients.

**Conclusion:**

Depressed patients may have low self-reported functioning due to emotional problems, pain, and their working ability may have decreased; however, the patients of both groups have an equal number of co-morbid diagnoses and their consultation rate is similar.

## Background

Patients with depressive disorder are common in family practice. About 10–18% of patients in primary care suffer from clinical depression [1-3].

Depression has been extensively studied in recent years. Several studies have shown that depressive disorder in primary care is associated with non-specific somatic symptoms, such as chronic pain and tiredness, and co-morbidity [4-6]. Also studies of patients with specific illnesses, e.g. cancer, diabetes mellitus, Parkinson's disease, and dementia, have shown higher rates of depression in comparison with patients without these disorders [7-9].

Depression often coexists with other psychiatric disorders. About half of patients with depression have co-morbid anxiety, and personality or alcohol use disorder [10].

Some authors have demonstrated that depression is associated with increased use of medical services, which can be partly explained by associated medical conditions [11,12]. Furthermore, depression is also one of the leading causes of disability in the world [13].

Several studies have shown that depressed patients have lower social and physical functioning and poorer health related quality of life than patients without depression [11,14,15]. However, it remains unclear whether depressed patients indeed have poorer health than non-depressed patients, or whether this is only related to their perception of having poor health [14,15].

The aims of this study were to find out (1) how depressed patients themselves evaluate their health; (2) which co-morbid diseases are associated with depression; (3) how depression influences the patients' consultation rate in family practice; (4) how disability is associated with depression; and (5) how depression influences the patients' working ability.

## Methods

The current study is part of the PREDICT study carried out in 2003–2005 in 23 family practices across Estonia (15 in urban and 8 in rural areas) [16-18]. The study design has been described in detail elsewhere [16,18]. The doctors were instructed to recruit patients according to the criteria of the project [16,18]. The inclusion criteria were: consecutive attendees of family doctor's consultations; patients aged 18 to 75 years; patients from urban and rural areas. The exclusion criteria were non-Estonian speakers, and presence of a severe organic mental or terminal illness [16,18].

After the participants had given their informed consent, a detailed interview was carried out, either at their home or at family practice centres, by specially trained interviewers within two weeks. Occurrence of major depression was assessed using the Depression Section of the Composite International Diagnostic Interview (CIDI) version 2.1, which provides a six-month depression diagnosis according to the International Classification of Diseases (ICD-10) [19]. Additionally, every patient filled in questionnaires for assessment of sociodemographic and health-related risk factors for depression [16]. Among the questionnaires used in our study was the SF-12 Health Survey (Version 1.0), which is among the most widely used instruments to assess the patients' functioning and to measure the patients' self-evaluation of their mental and physical health, and health-related quality of life [20].

To study the co-morbidity and healthcare utilization of the depressed and the non-depressed patients, we asked relevant information from their family doctors. We sent registration forms to the family doctors inquiring about the patients' co-morbid diseases by the ICD-10, number of visits to the family doctor and number of days on sick-leave due to all causes between January 2003 and December 2005, and disability. The doctors were asked to fill in the registration forms using information from the patients' medical records. All registration forms distributed among the family doctors were returned.

Disability was defined in case the patient had some somatic or mental disease that limited working and he/she had the right to receive social benefits from the social system.

Days on sick-leave were defined as days lost from work due to some illness.

### Statistics

The Statistical Package for the Social Sciences (SPSS) for Windows Release 10.0.1 [21] was used for data analysis.

Standard methods (mean, standard deviation, percentages) were used for descriptive statistics. Differences between the depressed and the non-depressed patients were analysed with the Chi-Square Test and the t-test. To find out the factors associated independently with depression, we used logistic regression analysis and computed the odds ratios (OR) with 95% confidence intervals (95% CI). In a logistic regression model, we combined and tested the variables that associated with depression, such as gender; disability; patient's self-evaluation of health in general; less accomplishment as a result of problems of physical health; limitation in work due to problems of physical health; less accomplishment as a result of emotional problems; working less carefully than usual as a result of emotional problems; having pain that interferes with normal work; health-related limitation in regular activities as moving a table or climbing stairs; and social activities interfered due to health problems; co-morbid endocrine, nutritional and metabolic disease; co-morbid mental or behavioural disorder; and number of days on sick-leave.

All tests were two-sided and statistical significance was assumed when p < 0.05.

### Ethics

The Ethics Committee of the University of Tartu approved the study.

## Results

**Figure 1:**
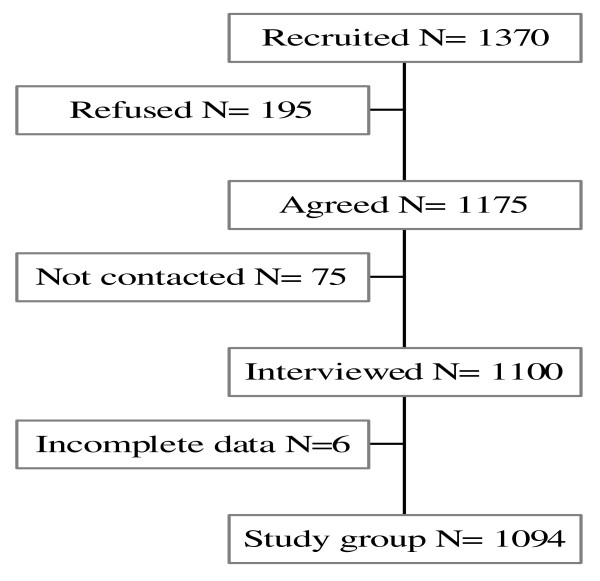
**Flowchart of the study population**.

Figure 1 shows the flowchart of the study population.

The family doctors recruited 1370 patients: 195 of them declined, making contact with 75 of the patients failed, and 6 interviews were excluded due to incomplete data. Thus the final study group consisted of 1094 patients.

### Depression and sociodemographic factors

The demographic data of the study group are presented in Table 1.

**Table 1:** Descriptive characteristics of male and female patients with or without depression assessed by the CIDI

**Characteristics**	**Total**	**Non-depression**	**Depression**	
	**n (%)**	**n (%)**	**n (%)**	**p-value**
Total	1094	864 (79)	230 (21)	
**Gender**				0.001 ^a^
Male	291 (27)	249 (86)	42 (14)	
Female	803 (73)	615 (77)	188 (23)	
**Place of residence**				0.794^a^
Rural area	263 (24)	206 (78)	57 (22)	
Urban area	813 (76)	644 (79)	169 (21)	
**Age **(mean ± SD)				
Total	45.5 ± 15.7	45.6 ± 16.0	44.4 ± 13.6	0.179 ^b^
Male	46.4 ± 16.1	46.8 ± 16.4	44.2 ± 13.8	
Female	45.0 ± 15.4	45.2 ± 15.9	44.4 ± 13.6	
**Disability**				0.034 ^a^
Yes	199 (18)	146 (73)	53 (27)	
No	882 (82)	708 (80)	174 (20)	

^a^Differences were analysed by the Chi-squared test.

^b^Differences were analysed by the t-test.

A six-month depressive episode occurred in 230 (21%) patients. Significantly more female patients were depressed than male patients: of the male patients 14% and of the female patients 23% were depressed (p = 0.001).

Place of residence in the rural or in the urban area/region did not influence occurrence of depression in our study (p = 0.794).

The mean age of depressed patients was 44.4 years, while there was no statistical difference between the mean age of male (44.2) and female (44.4) depressed patients (p = 0.179).

Depression was more common in patients with disability: 27% of the patients with disability versus 20% of the patients without disability were depressed during the study (p = 0.034).

### Depression and patients' self-evaluation of their health

Table 2 presents the answers of depressed and non-depressed patients to the SF-12.

**Table 2:** Depressed and the non-depressed patients' self-evaluation of their health assessed by the SF-12

**Answers**	**Non-depression**	**Depression**	**p-value**
	**n (%)**	**n (%)**	
**Health in general**			
Excellent	25 (3)	3 (1)	0.000 ^a^
Very good	139 (16)	21 (9)	
Good	289 (34)	53 (23)	
Fair	302 (35)	101 (45)	
Poor	106 (12)	50 (22)	
**Accomplished less as a result of problems of physical health**	389 (45)	154 (67)	0.000 ^a^
**Limitation in work as a result of problems of physical health**	423 (49)	165 (72)	0.000 ^a^
**Accomplished less as a result of emotional problems**	396 (46)	172 (75)	0.000 ^a^
**Less careful than usual as a result of emotional problems**	301 (35)	141 (61)	0.000 ^a^
**Normal work interfered with by pain**			
Not at all	283 (33)	43 (19)	0.000 ^a^
Slightly	262 (30)	59 (26)	
Moderately	130 (15)	37 (16)	
Quite a bit	135 (16)	49 (21)	
Extremely	53 (6)	42 (18)	
**Limitations as moving a table due to health**			
Limited a lot	47 (5)	21 (9)	0.005 ^a^
Limited a little	168 (20)	61 (27)	
Not limited at all	649 (75)	148 (64)	
**Limitations in climbing several stairs due to health**			
Limited a lot	68 (8)	27 (12)	0.023 ^a^
Limited a little	231 (27)	74 (32)	
Not limited at all	565 (65)	128 (56)	
**Social activities interfered with by health**			
All of the time	23 (3)	12 (5)	0.000 ^a^
Most of the time	94 (11)	43 (19)	
Some of the time	201 (23)	57 (25)	
A little of the time	214 (25)	61 (26)	
None of the time	332 (38)	57 (25)	

^a^Differences were analysed by the Chi-squared test.

We found that 22% of the depressed patients and 12% of the non-depressed patients evaluated their health in general as poor and 1% of the depressed and 3% of the non-depressed patients reported that their health in general was excellent (p = 0.000).

Compared with non-depressed patients depressed patients reported much more limitations in their work and significantly less accomplishment owing to problems of physical and mental health during the past four weeks (p = 0.000).

According to our analysis, depressed patients evaluated that pain interfered more with their normal work in comparison with non-depressed patients: 18% of the depressed patients and 6% of the non-depressed patients described that pain had extremely interfered with their normal work during the past four weeks (p = 0.000).

Depressed patients reported more limitations in such physical activities as moving a table or climbing stairs compared with non-depressed patients.

Of the depressed patients 24% and of the non-depressed patients 14% reported that their social activities were distributed by health problems all of the time or most of the time (p = 0.000).

### Depression and co-morbidity

The four most prevalent causes to consult the family doctor for all patients were diseases of the musculoskeletal, respiratory and cardiovascular systems. Mental and behavioural disorders (not depression) occupied the ninth place among the causes to consult the family doctor (Table 3).

**Table 3:** Depression and prevalence of co morbid diagnoses by the ICD-10

**Diagnosis by ICD-10**	**Total number of cases** n	**Non-depression** n (% of non-depressed)	**Depression** n (% of depressed)	**p-value**
Diseases of the musculoskeletal system and the connective tissue	483	384 (45)	99 (44)	0.707 ^a^
Diseases of the respiratory system	460	368 (43)	92 (41)	0.497 ^a^
Diseases of the cardiovascular system	395	316 (37)	79 (35)	0.587 ^a^
Diseases of the digestive system	196	158 (17)	38 (17)	0.562 ^a^
Endocrine, nutritional and metabolic diseases	151	129 (15)	22 (10)	0.040 ^a^
Diseases of the genitourinary system	144	105 (12)	39 (17)	0.062 ^a^
Certain infectious and parasitic diseases	139	118 (14)	21 (9)	0.074 ^a^
Diseases of the skin and subcutaneous tissue	135	113 (13)	22 (10)	0.175 ^a^
Mental and behavioural disorders	119	85 (10)	34 (15)	0.042 ^a^
Diseases of the nervous system	94	70 (8)	24 (11)	0.289 ^a^
Symptoms, signs and abnormal clinical and laboratory findings not classified elsewhere	93	67 (8)	26 (11)	0.109 ^a^
Diseases of the ear and mastoid process	65	53 (6)	12 (5)	0.753 ^a^
Injury, poisoning and certain other consequences of external causes	51	41 (5)	10 (4)	1.000 ^a^
Neoplasms	39	30 (3)	9 (4)	0.693 ^a^
Any co-morbid diagnosis	960	754 (87)	206 (90)	0.368 ^a^

^a^Differences were analysed by the Chi-squared test.

Table 3 shows that 90% of the depressed patients and 87% of the non-depressed patients had at least one co-morbid diagnosis (p = 0.368).

**Figure 2:**
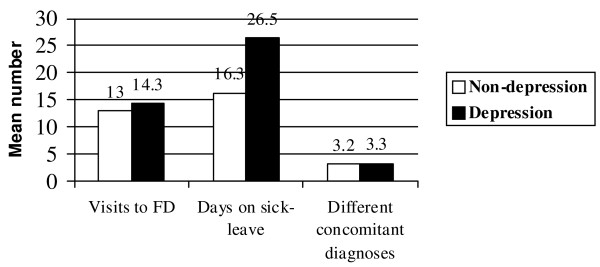
**Mean number of visits to the FD, days on sick-leave and different co-morbid diagnoses of patients with non-depression and patients with depression**. Differences were analysed by the t-test. Mean number of visits to the FD, p = 0.156. Mean number of days on sick-leave, p = 0.002. Mean number of different co-morbid diagnoses, p = 0.546.

There were no significant differences between the mean number of different co-morbid diagnoses for depressed and non-depressed patients (p = 0.546) (Figure 2) but depressed patients had significantly more co-morbid psychiatric disorders (F00-F99) (p = 0.042) and endocrine, nutritional and metabolic disorders (E00-E90) (p = 0.040) than non-depressed patients (Table 3).

### Depression and number of visits to the family doctor and days on sick-leave

Figure 2 illustrates associations between the patients' number of visits to the family doctor, number of days on sick-leave and number of different diagnoses by the ICD-10.

Depressed patients consulted their family doctor slightly more than non-depressed patients but the difference was not significant (p = 0.156).

However, depressed patients were significantly longer on sick-leave compared with non-depressed patients (26.5 and 16.3 days, respectively) (p = 0.002).

### Depression and factors associated with depression in logistic regression analysis

In logistic regression analysis four variables were independently associated with depression. These were less accomplishment as a result of emotional problems (OR 1.80; 95% CI 1.18–2.72), being less careful than usual as a result of some emotional problems (OR 1.81; 95% CI 1.26–2.60), having pain that extremely interfered with work (OR 2.50; 95% CI 1.33–4.70), and being more days on sick-leave (OR 1.00; 95% CI 1.00–1.01) (Table 4).

**Table 4:** Association of different variables with depression in logistic regression analysis

**Variable**	**Odds ratio (OR)**	**95% confidence interval (95% CI)**
**Accomplished less as a result of emotional problems (SF-12)**		
**Yes**	**1.80**	**1.18–2.72**
No	1.0	
**Less careful than usual as result of any emotional problems (SF-12)**		
**Yes**	**1.81**	**1.26–2.60**
No	1.0	
**Normal work interfered with by pain (SF-12)**		
Not at all	1.0	
Slightly	1.24	0.77–1.99
Moderately	1.23	0.70–2.17
Quite a bit	1.23	0.70–2.15
**Extremely**	**2.50**	**1.33–4.70**
**Days on sick-leave**	**1.00**	**1.00–1.01**

## Discussion

The main finding of this study is establishment of absence of association between depression and co-morbid diseases. Furthermore, analysis of the patients' medical records showed that depressed patients had as many concomitant diagnoses as non-depressed patients and they consulted the family doctor as often as non-depressed patients did.

To our knowledge, this is a new finding. Earlier studies have shown that co-morbidity is more common among depressed than non-depressed patients [1,22,23], and that depressed patients consult the doctor more frequently than non-depressed patients [1,18,24].

This discrepancy can be related to differences in the methodology used in the above studies and in our study. Firstly, the sources of information about co-morbidity and consultation rate were different: to prevent any information bias, we inquired the family doctors and not the patients about these variables [1,22,23]. Secondly, the instruments used to assess depression were also different: some studies employed screening instruments [22,23] and not diagnostic instruments such as CIDI. Thirdly, the length of the study period can also play a role: we registered all co-morbid diagnoses and visits to the family doctor for a period of three years while in other studies the pertinent period was only six months [18]. Indeed, depressed patients consult the doctor more often during an illness episode the duration of which is usually not more than six months so that after recovery, consultation rate will decrease. Cultural and organizational differences may also affect consultation rate. For example, in Estonia it is possible to consult the psychiatrist without referral. However, according to an earlier study, 98% of family doctors in Estonia treat depressed patients on their own [25]. Further studies are necessary to clarify all these aspects.

As there were no differences in the number of co-morbid diagnoses between depressed and non-depressed patients in our study, we can not claim that patients with co-morbid illnesses are more likely to have depression. Based on our findings, depression should be considered an independent serious disease that requires also an independent approach. Moreover, the impact of depression should not be underestimated when it occurs as a concomitant disease.

As almost all of our patients (88%) had some co-morbid diagnosis, irrespective of the fact whether they had depression or not, we can conclude that co-morbidity as a phenomenon is common in family practice. A similar prevalence of co-morbidity among primary care patients was found by Vuorilehto et al [5].

From the clinical point of view, it is important to point out that as almost all family practice attendees have some co-morbid diagnosis, their management is a challenge for the family doctor. According to van Weel et al., "co-morbidity is a regular feature of general practice and dealing with co-morbidity needs a patient-centred rather than a disease-oriented approach" [26].

In this study the factors that were separately associated with depression, such as gender, disability, health in general, limitations in work as a result of problems of physical health, social activities disturbed by health problems, co-morbid endocrine and mental disorders, were not associated with depression in a combined multifactor model.

Only four factors were independently associated with depression: less accomplishment as a result of emotional problems, being less careful than usual as a result of emotional problems, having pain that extremely interfered with work, and being more days on sick-leave.

Depressed patients reported that they accomplished less and were less careful as a result of emotional problems – these being typical emotional symptoms of depression.

Additionally, depressed patients reported having more pain than non-depressed patients. There is evidence that pain and depression are often associated [12,27]. Thus, from the practical point of view, it is important to acknowledge that, besides information about specific emotional symptoms, also information about non-specific symptoms such as pain should be obtained from depressed patients.

According to our results, depressed patients were more days on sick-leave than non-depressed patients. Previous studies have also shown that psychiatric problems are among the most common diagnostic groups accounting for high sickness absence [22,28].

It should be emphasised that the strength of our study is the fact that the study group was formed of consecutive general practice attendees but not simply of patients with emotional or psychiatric symptoms. A better knowledge of the problems of consecutive patients is important for the doctors' readiness to have necessary skills for management of patients with different problems in family practice.

The other strengths are a quite large and representative study group (1094 primary care patients), use of the reliable diagnostic instrument CIDI in assessment of depression, and the patients' and the family doctors' high motivation to contribute to the study (80% of the recruited patients completed the study).

The limitations of this study are lack of information about the reasons for being on sick-leave and about the number of days on sick-leave due to depression. On the other hand, establishment that depression is associated with high sickness absence seems more important than finding out specific reasons for being on sick-leave. In addition, the cross-sectional nature of the study makes it impossible to determine causality between depression and co-morbidity as well as between consultation rate and disability. However, our purpose was to compare the depressed and the non-depressed patients' co-morbidity, disability, and consultation rate rather than to study the mechanisms of the relationship.

## Conclusion

The results of this study show that depressed patients have low self-reported functioning related to emotional problems; they also report pain and their working ability is decreased. Yet, depressed patients consulted the family doctor as often as did patients without depression and the patients of both groups had an equal number of co-morbid diagnoses.

## Abbreviations

CIDI: Composite International Diagnostic Interview; FD: family doctor; FP: family practice; ICD-10: International Classification of Diseases.

## Competing interests

All authors declare that they have no competing interests related to the present study.

This study was financially supported by the Estonian Science Foundation (grants number 5696 and 7596) and by targeted financing (TARPO 0821).  

## Authors' contributions

KS participated in the designing of the study, collected and analysed the data and completed the manuscript. RK and HIM participated in the designing of the study, collected and analysed the data, and approved the final manuscript.

## Pre-publication history

The pre-publication history for this paper can be accessed here:


